# Trends and disparities in bone cancer mortality among US adults from 1999 to 2020: a joinpoint regression analysis based on the CDC WONDER database

**DOI:** 10.3389/fonc.2026.1718354

**Published:** 2026-03-05

**Authors:** Lei Wei, Yinghu Deng, Zhixiang Ma, Xiaosi Zhang

**Affiliations:** Department of Sports Medicine, Tongling People’s Hospital, Tongling, Anhui, China

**Keywords:** bone neoplasms, CDC WONDER, epidemiology, healthcare disparities, joinpoint regression, mortality, trends

## Abstract

**Objective:**

This study aims to describe trends in bone cancer-related mortality from 1999 to 2020 and analyze disparities across various demographic subgroups.

**Methods:**

Data were obtained from the CDC WONDER multiple cause of death database (1999–2020). Deaths of individuals aged 25 years or older with primary malignant bone cancer (ICD-10 codes C40–C41) as the underlying cause were included. AAMRs were calculated. Joinpoint regression analysis was employed to evaluate temporal trends and estimate the APC and AAPC.

**Results:**

From 1999 to 2020, a total of 25,859 bone cancer deaths were reported among US adults. The overall ASMR increased from 0.528 per 100,000 (95% CI: 0.495–0.562) in 1999 to 0.599 per 100,000 (95% CI: 0.569–0.682) in 2020, with an AAPC of 0.509 (95% CI: 0.196 to 0.931; p = 0.002) over the 22-year period. Joinpoint analysis identified three distinct segments: a non-significant decrease from 1999 to 2014 (APC = –0.218, p = 0.288), a significant increase from 2014 to 2018 (APC = 4.479, p = 0.157), and a non-significant decrease from 2018 to 2020 (APC = –1.779, p = 0.424). Mortality rates were higher in males (0.712 per 100,000) than in females (0.446 per 100,000). Adults aged 85 years and older had the highest mortality rate (3.430 per 100,000). Black or African American individuals experienced a higher mortality rate (0.584 per 100,000) compared to White (0.561 per 100,000) and Hispanic or Latino (0.335 per 100,000) individuals. Geographically, the South had the highest mortality rate (0.614 per 100,000), while the Northeast had the lowest (0.444 per 100,000).

**Conclusion:**

Bone cancer mortality among US adults showed an overall increasing trend from 1999 to 2020, with significant disparities by age, sex, race, and geographic region. The triphasic mortality trend may reflect evolving diagnostic technologies, treatment approaches, and healthcare accessibility. These findings provide valuable insights for public health planning and resource allocation aimed at reducing the burden of bone malignancies.

## 1 Introduction

Although malignant bone cancers account for less than 0.2% of all malignancies, they are highly aggressive and difficult to treat, often leading to severe functional impairment and high mortality rates, presenting a significant public health challenge (1). In the United States, approximately 3,500 new cases are diagnosed annually. The age distribution exhibits a bimodal pattern, with the first peak occurring in adolescents and young adults and the second in older adults, showing notable differences in pathological subtypes and prognosis across age groups (2, 3).

Over recent decades, the treatment paradigm has undergone profound transformation evolving from amputation alone to a multidisciplinary approach incorporating neoadjuvant chemotherapy, limb-salvage surgery, radiation therapy, and increasingly, targeted therapies. These advances have significantly improved survival outcomes, particularly among children and adolescents (4, 5). However, whether these therapeutic benefits have translated into long-term population-level mortality decline, especially among adult patients, remains unclear due to limited robust evidence from large national datasets.

Existing research on bone cancer survival and mortality has important limitations. Most studies have focused on children, adolescents, or specific histological subtypes such as osteosarcoma or Ewing sarcoma, lacking comprehensive assessment across all adult age groups (6, 7). Many reports are based on single-institution experiences or regional cancer registries (e.g., the SEER database), which may limit generalizability (8, 9). Although significant racial, socioeconomic, and geographic disparities in bone cancer outcomes are well-documented, the dynamic trends in these health inequalities over the past two decades remain poorly understood (10). A critical question persists: whether survival disadvantages among non-Hispanic Black individuals, residents of low socioeconomic status areas, and those in rural communities have persisted, widened, or narrowed essential information for designing targeted public health interventions (11).

The CDC WONDER (Wide-ranging Online Data for Epidemiologic Research) database provides a comprehensive and publicly accessible national mortality registry, offering a unique resource for systematically examining long-term trends in rare diseases and associated health inequities (12). With coverage of all U.S. counties, it enables detailed stratified analyses across multiple dimensions. The Joinpoint regression model is a powerful statistical tool that can identify inflection points in mortality trends and quantify annual percent change over specific periods, thereby revealing the potential impacts of public health events such as policy changes and treatment advances (13).

Therefore, this study aims to use the authoritative CDC WONDER database to conduct a comprehensive analysis of bone cancer-related mortality from 1999 to 2020 among adults aged 25 years and older in the United States. We will describe overall temporal trends and examine differentials by key demographic dimensions including sex, age, race/ethnicity, and geographic region. The findings are expected to yield crucial evidence for evaluating the effectiveness of current prevention and treatment strategies, identifying persistently high-risk populations, uncovering dynamic health inequalities, and informing future resource allocation and precision public health planning.

## 2 Materials and methods

### 2.1 Data source

This study utilized publicly available data from the CDC WONDER(https://wonder.cdc.gov/) Multiple Cause of Death database (1999–2020). The CDC WONDER system compiles mortality data derived from all death certificates in the United States, capturing over 2.8 million deaths annually. We extracted records of individuals aged 25 years or older from all 50 states and the District of Columbia whose underlying cause of death was classified as malignant neoplasms of bone and articular cartilage (ICD-10 codes: C40–C41). Code C40 corresponds to malignancies of long bones and articular cartilage of the limbs, while C41 includes those occurring in other and unspecified sites, such as the jaw, vertebrae, ribs, sacrum, and pelvic bones. As this study relied exclusively on publicly available, anonymized, and aggregated data from the CDC WONDER database which contains no personally identifiable information it was exempt from institutional review board approval.

### 2.2 Case selection and classification

All death records from January 1, 1999, to December 31, 2020, were extracted using the following selection criteria: Underlying cause of death: ICD-10 codes C40–C41 (malignant neoplasms of bone and articular cartilage);Age group: 25 years and older (adults);Place of residence: All 50 U.S. states and Washington D.C. Demographic variables included: Sex: Male, Female; Age group: 25–34, 35–44, 45–54, 55–64, 65–74, 75–84, ≥85 years; Race/Ethnicity: Black or African American, White, Hispanic or Latino; U.S. Census region: Northeast, Midwest, South, West.

### 2.3 Statistical analysis

Crude mortality rates and AAMR were calculated for each year from 1999 to 2020. AAMR was computed using the direct standardization method based on the year 2000 U.S. standard population. Mortality rates are expressed per 100,000 population, along with their corresponding 95%CI.Temporal trends in mortality were analyzed using the Joinpoint Regression Program (Version 4.9.1.0) developed by the National Cancer Institute (13). This model identifies significant change points (joinpoints) in trends and calculates the APC for each segment, as well as the AAPC over the entire period, both with 95% CIs. Statistical significance was assessed using the Monte Carlo permutation method, with a p-value < 0.05 considered statistically significant. The maximum number of joinpoints was set to 3, with a minimum of 4 data points between change points.

All analyses were stratified by sex, age group, and race/ethnicity. Due to the low number of annual cases (<20 per year) among Hispanic or Latino individuals, only overall trends are reported for this group without joinpoint analysis. In accordance with the CDC WONDER data use agreement, mortality counts fewer than 10 per year are suppressed to protect privacy; such years were treated as missing values in the trend analysis.

## 3 Results

### 3.1 Overall characteristics of death from malignant bone cancer

Between 1999 and 2020, a total of 25859 deaths from malignant bone cancer were reported among adults in the United States. Males accounted for 55.54% (14363 cases) of these deaths, while females comprised 44.46% (11496 cases).Analysis by age group revealed that individuals aged 75 years and older represented the largest proportion of cases (37.30%, 9646 cases), followed by those aged 65–74 (19.68%, 5,088 cases), 55–64 (15.74%, 4070 cases), 25–44 (16.15%, 4176 cases), and 45–54 (11.13%, 2879 cases).By race/ethnicity, the majority of deaths occurred among White individuals (85.95%, 22226 cases), followed by Black or African American (10.99%, 2,841 cases) and Hispanic or Latino (3.06%, 792 cases) individuals. Geographically, the Southern U.S. region had the highest number of deaths (42.07%, 10878 cases), followed by the West (21.45%, 5547 cases), the Midwest (20.93%, 5411 cases), and the Northeast (15.56%, 4023 cases) (**Table 1**; **Supplementary Table 1**).

**Table 1:** Demographic characteristics of deaths due to Bone Cancer in the United States from 1999 to 2020.

Variables	Deaths	Population	AAMR(95%CI)
Overall	25859	4473854489	0.538(0.531-0.544)
Sex			
Male	14363	2154556911	0.712(0.700-0.723)
Female	11496	2319297578	0.446(0.437-0.454)
Race/Ethnicity			
Hispanic or Latino	792	295429555	0.335(0.311-0.359)
Black or African American	2841	546447527	0.584(0.562-0.606)
White People	22226	3631977407	0.561(0.554-0.68)
Census region			
Northeast	4023	827193779	0.444(0.430-0.458)
Midwest	5411	969567311	0.527(0.513-0.542)
South	10878	1652256217	0.614(0.602-0.626)
West	5547	1024837182	0.531(0.517-0.545)
Urbanization			
Metropolitan(Urban)			
Nonmetropolitan(Rural)	5560	678634169	0.698(0.679-0.717)
Ten-Year Age Groups	20299	3795213822	0.511(0.504-0.518)
25-34 years	2259	920089469	0.246(0.235-0.256)
35-44 years	1917	931287288	0.206(0.197-0.215)
45-54 years	2879	927576220	0.310(0.299-0.322)
55-64 years	4070	766424847	0.531(0.5155-0.547)
65-74 years	5088	510458341	0.997(0.969-1.024)
75-84 years	5547	298504433	1.858(1.809-1.907)
85+ years	4099	119513891	3.430(3.325-3.535)

AAMR, age‐adjusted mortality rate; CI, confidence interval.

### 3.2 Age-standardized mortality trends

Between 1999 and 2020, the AAMR for malignant bone cancer in the general population increased from 0.528 per 100,000 (95% CI: 0.495–0.562) in 1999 to 0.599 per 100,000 (95% CI: 0.569–0.682) in 2020. Joinpoint regression analysis revealed an AAPC of 0.509 (95% CI: 0.196 to 0.931) over the 22-year period, indicating a statistically significant overall upward trend in mortality (p = 0.002) (**Tables 1**, **2**; **Supplementary Figures 1**, **2**).

**Table 2:** Annual percentage changes and average annual percentage changes in bone cancer in the USA from 1999 to 2020.

Variables	Trend segment	Year interval	APC (95% CI)	AAPC (95% CI)	*P-*value
Entire Cohort	–	1999-2020	–	0.509(0.196-0.931)	0.002
	1	1999-2014	-0.218(-1.075 to 1.640)	–	0.288
	2	2014-2018	4.479(-2.104 to 7.090)	–	0.157
	3	2018-2020	-1.779(-5.355 to 2.868)	–	0.424
Sex					
Male	–	1999-2020	–	0.881(0.369 to 1.344)	0.001
	1	1999-2013	-0.204(-2.239 to 0.470)	–	0.498
	2	2013-2020	3.087(1.465 to 8.385)	–	<0.001
Female	–	1999-2020	–	0.638(-0.126 to 1.393)	0.096
	1	1999-2013	-0.390(-6.171 to 2.852)	–	0.296
	2	2013-2020	2.726(0.392 to 10.670)	–	0.036
Race/Ethnicity					
Black or African American	–	1999-2020	–	0.750(0.067 to 1.545)	0.034
White	–	1999-2020	–	0.946(0.690 to 1.360)	<0.001
	1	1999-2005	1.204(-0.323 to 5.091)	–	0.083
	2	2005-2009	-2.870(-5.474 to 2.396)	–	0.079
	3	2009-2020	2.229(1.508 to 3.238)	–	0.023
Census region					
Northeast	–	1999-2020	–	0.284(-0.521 to 1.174)	0.418
Midwest	–	1999-2020	–	0.157(-0.336 to 0.696)	0.481
South	–	1999-2020	–	0.788(0.070 to 1.505)	0.033
	1	1999-2012	-0.382(-5.946 to 0.845)	–	0.386
	2	2012-2020	2.718(0.794 to 10.020)	–	0.021
West	–	1999-2020	–	1.171(0.570 to 1.757)	<0.001
	1	1999-2011	-0.003(-4.494 to 0.987)	–	0.833
	2	2011-2020	2.757(1.539 to 7.628)	–	0.006
Urbanization					
Metropolitan(Urban)	–	1999-2020	–	0.834(0.399 to 1.243)	<0.001
	1	1999-2013	-0.090(-1.985 to 0.530)	–	0.681
	2	2013-2020	2.705(1.257 to 7.503)	–	0.001
Nonmetropolitan(Rural)	–	1999-2020	–	0.268(-0.149 to 0.722)	0.199

Further analysis identified three distinct temporal segments based on significant change points:1999–2014: Mortality showed a non-significant decrease, with an APC of –0.218 (95% CI: –1.075 to 1.640, p=0.288).2014–2018: Mortality increased at an APC of 4.479 (95% CI: –2.104 to 7.090, p =0.157), though this change was not statistically significant.2018–2020: Mortality declined again, with an APC of –1.779 (95% CI: –5.355 to 2.868, p = 0.424), which was also not statistically significant (**Table 2**; **Supplementary Figure 1**).

### 3.3 Mortality trends stratified by demographic characteristics

#### 3.3.1 Gender differences

**Figure 1:**
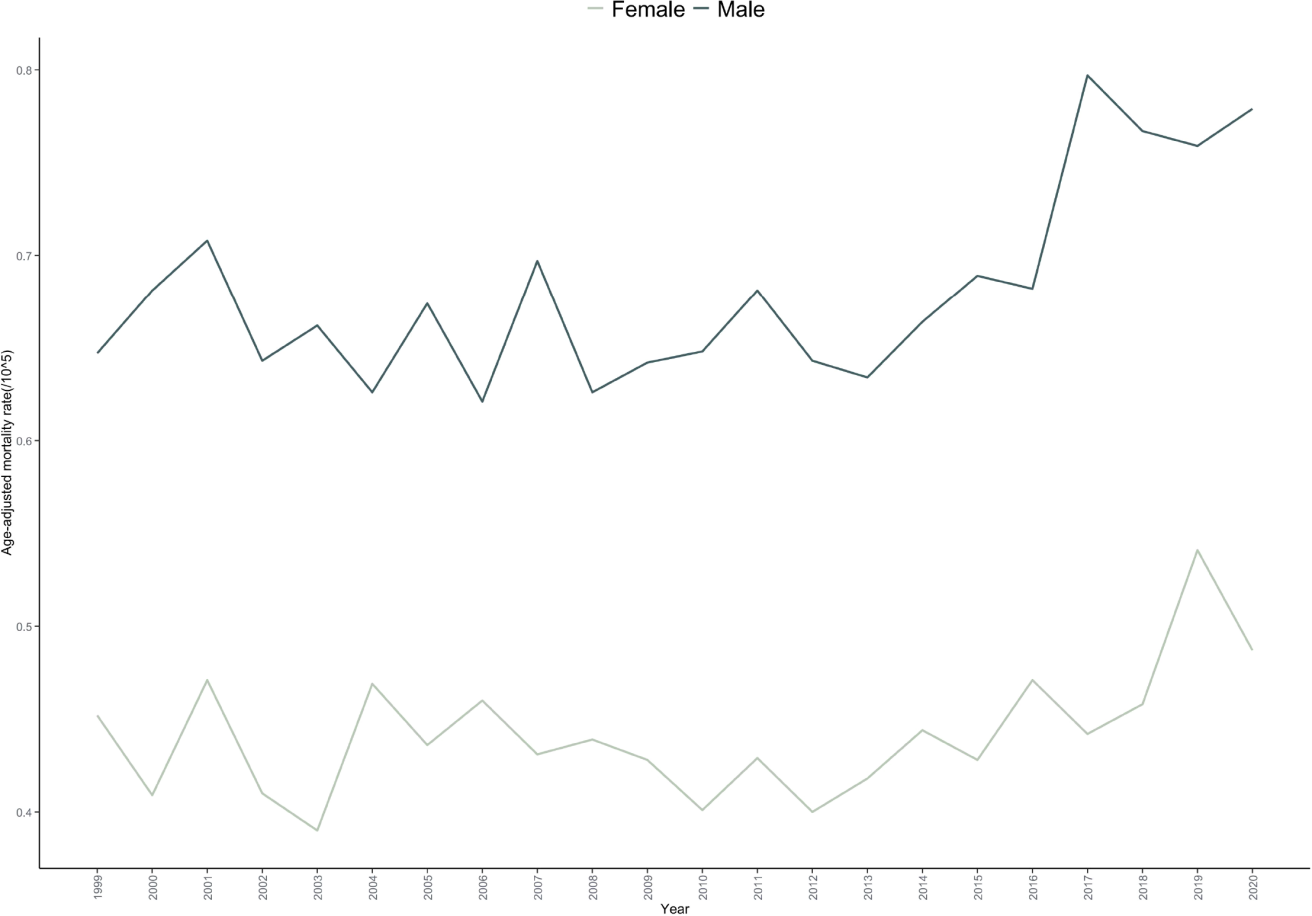
Trends in age-adjusted mortality rate for bone cancer-related mortality in the United States(1999-2020), stratified by gender.

The AAMR for malignant bone cancer was higher in males (0.712 per 100,000, 95% CI: 0.700–0.723) than in females (0.446 per 100,000, 95% CI: 0.437–0.454). Both sexes exhibited an overall increase in mortality over the study period, with an AAPC of 0.881 (p = 0.001) in males and 0.638 (p=0.096) in females. Joinpoint analysis revealed distinct temporal trends: Among males, mortality showed a non-significant decline from 1999 to 2013 (APC = –0.204, p=0.498), followed by a significant increase from 2013 to 2020 (APC = 3.087, p < 0.001).Among females, a non-significant gradual decrease occurred from 1999 to 2013 (APC=–0.390, p=0.296), after which mortality rose significantly from 2013 to 2020 (APC = 2.726, p=0.036) (**Tables 1**, **2**, **Figure 1**; **Supplementary Table 2** and **Supplementary Figure 3**).

#### 3.3.2 Age group differences

Mortality rates exhibited a clear gradient increasing with age. The highest AAMR was observed in the ≥85 years group (3.430 per 100,000; 95% CI: 3.325–3.535), followed by the 75–84 years group (1.858 per 100,000), 65–74 years group (0.997 per 100,000), 55–64 years group (0.531 per 100,000), 45–54 years group (0.310 per 100,000), 25–34 years group (0.246 per 100,000), and the 35–44 years group (0.206 per 100,000) (**Table 1**).

#### 3.3.3 Ethnic differences

**Figure 2:**
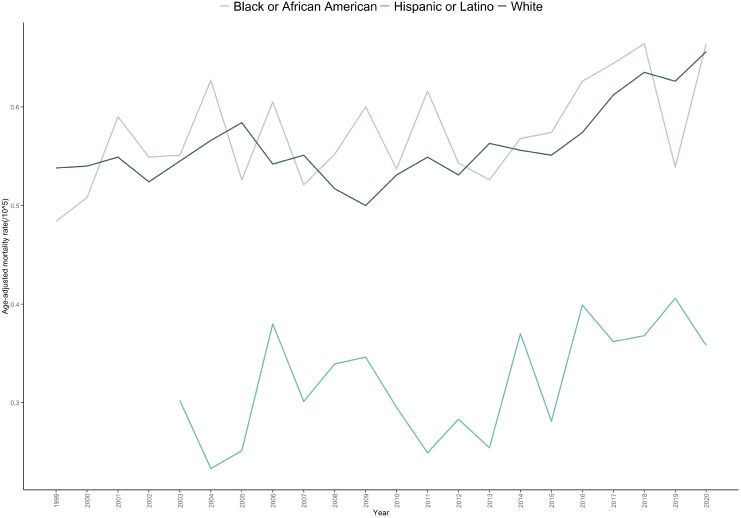
Trends in age-adjusted mortality rate for bone cancer-related mortality in the United States (1999-2020), stratified by race.

The AAMR was highest among Black or African American individuals (0.584 per 100,000; 95% CI: 0.562–0.606), followed by White individuals (0.561 per 100,000; 95% CI: 0.554–0.568) and Hispanic or Latino individuals (0.335 per 100,000; 95% CI: 0.311–0.359).Distinct mortality trends were observed across racial/ethnic groups: Black or African American: A sustained increasing trend was observed, with an AAPC of 0.750 (p=0.034).White people: Mortality increased non-significantly from 1999 to 2005 (APC = 1.204, p=0.083),declined between 2005 and 2009 (APC=–2.870, p=0.079), and then rose significantly from 2009 to 2020 (APC = 2.229, p=0.023).Hispanic or Latino: Due to the limited number of cases, only the overall trend is reported; joinpoint analysis was not performed (**Tables 1**, **2**, **Figure 2**; **Supplementary Table 3** and **Supplementary Figure 4**).

#### 3.3.4 Geographical differences

**Figure 3:**
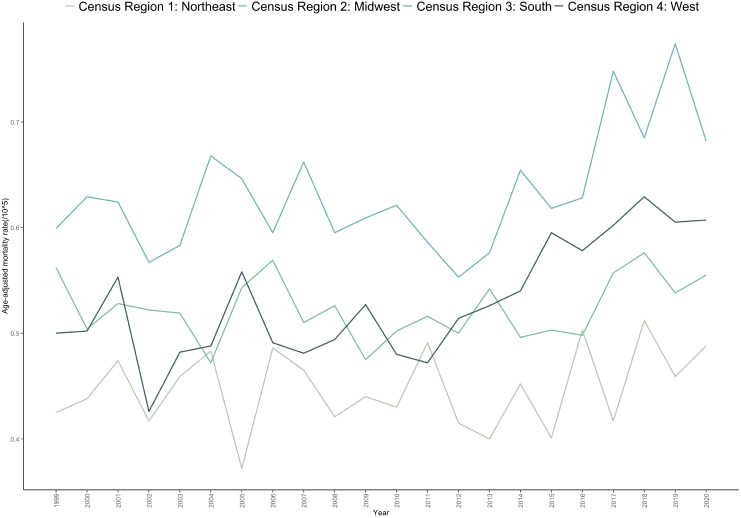
Trends in age-adjusted mortality rate for bone cancer-related mortality in the United States (1999-2020), stratified by region.

Analysis by U.S. Census region showed that the South had the highest age-adjusted mortality rate (AAMR) at 0.614 per 100,000 (95% CI: 0.602–0.626), followed by the West (0.531 per 100,000), the Midwest (0.527 per 100,000), and the Northeast (0.444 per 100,000).Mortality trends varied by region: Northeast: A non-significant increasing trend was observed, with an AAPC of 0.284 (p = 0.418).Midwest: A non-significant overall increase occurred, with an AAPC of 0.157 (p=0.481).South: A significant overall increase was found (AAPC = 0.788, p=0.033), characterized by a non-significant decline from 1999 to 2012 (APC=–0.382, p=0.386), followed by a significant increase from 2012 to 2020 (APC = 2.718, p=0.021).West: A significant upward trend was observed (AAPC = 1.171, p < 0.001), with a non-significant decrease from 1999 to 2011 (APC = –0.003, p = 0.833) and a significant increase from 2011 to 2020 (APC = 2.757, p=0.006) (**Tables 1**, **2**, **Figure 3**; **Supplementary Table 4** and **Supplementary Figure 5**).

### 3.4 Mortality trends stratified by urbanization level

**Figure 4:**
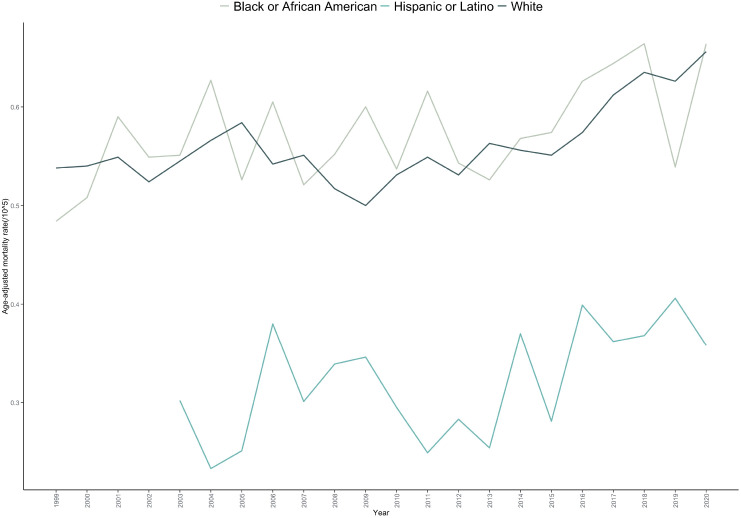
Trends in age-adjusted mortality rate for bone cancer-related mortality in the United States (1999-2020), stratified by metropolitan status.

Residents of non-metropolitan areas had a higher age-adjusted mortality rate (AAMR = 0.698 per 100,000) for bone malignant tumors compared to those in metropolitan areas (AAMR = 0.511 per 100,000).Trend analysis revealed distinct patterns by residential area: Non-metropolitan areas: exhibited a steady increasing trend in mortality, with an AAPC of 0.268.Metropolitan areas: also showed an overall upward trend (AAPC = 0.834),characterized by a non-significant decline from 1999 to 2013 (APC=–0.090, p=0.681), followed by a significant increase from 2013 to 2020 (APC = 2.705, p = 0.001) (**Tables 1**, **2**, **Figure 4**; **Supplementary Table 5** and **Supplementary Figure 6**).

### 3.5 Mortality Trends Stratified by States

**Figure 5:**
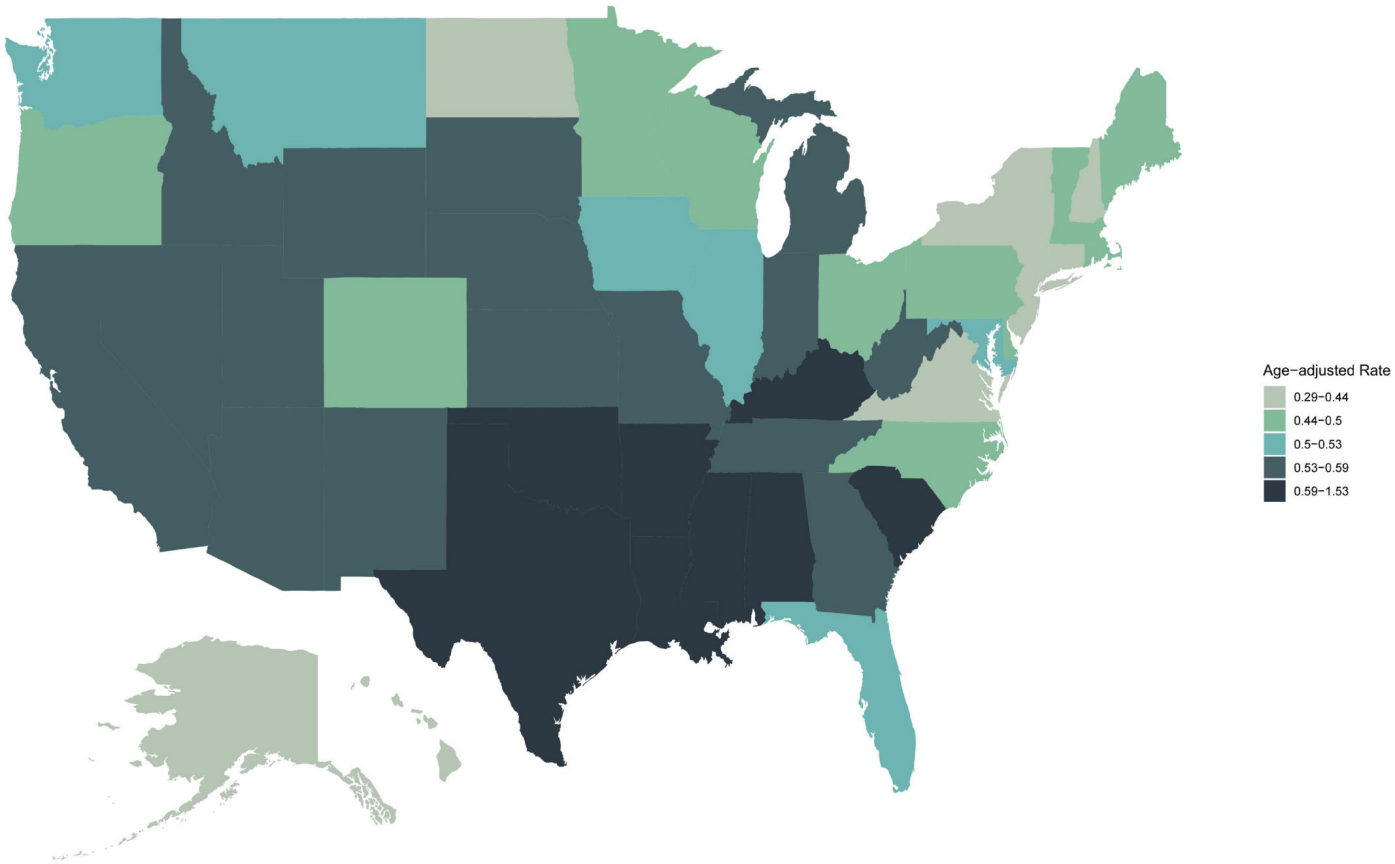
Trends in age-adjusted mortality rate for bone cancer-related mortality in the United States (1999-2020).

Significant geographic variation in bone malignant tumor mortality was observed across U.S. states. Mississippi recorded the highest age-adjusted mortality rate (AAMR = 1.355 per 100,000; 95% CI: 1.294–1.416), followed by Arkansas (AAMR = 1.142; 95% CI: 1.043–1.242), Louisiana (AAMR = 0.950; 95% CI: 0.875–1.026), Alabama (AAMR = 0.897; 95% CI: 0.828–0.966), and Oklahoma (AAMR = 0.776; 95% CI: 0.702–0.850). Many of these states are located in the South. In contrast, the lowest AAMR was observed in the District of Columbia (0.286; 95% CI: 0.188–0.416), followed by Connecticut (0.390; 95% CI: 0.340–0.440) and North Dakota (0.392; 95% CI: 0.282–0.502) (**Figure 5**; **Supplementary Table 6**).

## 4 Discussion

This comprehensive analysis of bone malignant tumor mortality trends among U.S. adults from 1999 to 2020 using the nationally representative CDC WONDER database reveals several critical public health insights. Our findings demonstrate a statistically significant overall increasing trend in age-adjusted mortality rates (AAPC = 0.509%), substantial and persistent health inequities across demographic and geographic subgroups, and a pivotal inflection point in the early 2010s that shifted mortality trajectories upward across nearly all populations. These patterns have important implications for understanding the epidemiological evolution of bone malignancies, evaluating treatment strategies, and guiding future public health interventions.

Population aging emerges as the primary driver of increasing mortality trends. Our data indicate that patients aged ≥75 years accounted for 37.30% of all deaths, exhibiting an age-adjusted mortality rate of 3.430 per 100,000 significantly higher than younger groups. Older adults are predominantly affected by histological subtypes with poor chemotherapy responses, including chondrosarcoma and high-grade undifferentiated pleomorphic sarcoma (14). Additionally, multiple comorbidities, compromised performance status, and reduced organ reserve often limit their tolerance for radical surgery or intensive chemotherapy, resulting in suboptimal treatment and diminished therapeutic effectiveness (15, 16). The continued expansion of the oldest age groups directly contributes to rising overall mortality rates.

Evolving diagnostic practices and death certification standards have also influenced observed trends. Advanced imaging techniques such as computed tomography, magnetic resonance imaging, and positron emission tomography have become increasingly widespread over the past two decades (17). This has improved detection of bone malignancies in anatomically complex areas or those with indolent growth, which might previously have been identified only at autopsy. Concurrently, improvements in cause-of-death reporting accuracy may have led to more deaths being correctly attributed to bone malignancies, contributing to increased case ascertainment that manifests statistically as rising mortality rates.

Treatment limitations remain significant despite therapeutic advances. While multidisciplinary approaches have revolutionized management of childhood and adolescent osteosarcoma and Ewing sarcoma (4), these successes have not translated equally into survival benefits for adult and elderly patients. Effective second-line therapies remain severely limited for those with metastatic, recurrent, or refractory disease (18). Although targeted therapies and immune checkpoint inhibitors offer promise, their applicability and duration of efficacy remain limited (19, 20). This therapeutic imbalance means that a large and growing population of adult patients has not fully benefited from recent medical advances. The upward trend in overall bone malignant tumor mortality contrasts sharply with improved survival reports from studies focusing on children, adolescents, or specific pathological subtypes (21, 22), reflecting the complex interplay of demographic shifts, diagnostic evolution, and therapeutic limitations affecting predominantly adult populations.

Our analysis confirms that mortality burden is heavily concentrated among socioeconomically and geographically disadvantaged groups, revealing profound health inequities. Racial disparities remain significant, with the age-adjusted mortality rate among Black or African American residents (0.584 per 100,000) consistently higher than White residents (0.561 per 100,000). These disparities reflect systemic barriers including inadequate insurance coverage, limited access to specialized sarcoma centers, later-stage diagnosis, healthcare system distrust, and lower clinical trial enrollment (23, 24).

Geographic disparities are equally concerning. Southern U.S. mortality rates (0.614 per 100,000) significantly exceed other regions, with Mississippi (1.355 per 100,000), Arkansas (1.142 per 100,000), and Alabama (0.897 per 100,000) representing particularly high-burden areas within the “Cancer Belt” (25). These regions are characterized by higher poverty rates, lower educational attainment, higher uninsured rates, fewer medical resources, and higher prevalence of unhealthy behaviors, creating conditions that hinder effective cancer prevention, early detection, and treatment (26, 27). Rural-urban disparities represent another critical dimension of health inequality. Non-metropolitan residents experienced substantially higher mortality (0.698 per 100,000) than metropolitan residents (0.511 per 100,000). Geographic isolation creates major access barriers including long travel distances to specialized centers, high transportation costs, lost wages, and challenges attending follow-up visits (28, 29). These obstacles often result in delayed diagnosis, reduced treatment adherence, and interrupted care, all adversely affecting survival outcomes.

Joinpoint regression analysis revealed a critical and concerning shift in mortality trends: after a period of slow decline or relative stability from 1999 to approximately 2013, mortality rates began a consistent and statistically significant accelerated increase across nearly all subgroups starting around 2013 (e.g., APC = 4.479% for the total population during 2014–2018; APC = 3.087% for males during 2013–2020).While not all temporal segments achieved statistical significance, it is important to consider the potential clinical and epidemiological meaning of these trends, as statistical non-significance does not necessarily imply a lack of clinical relevance, particularly in the context of rare diseases where smaller case numbers may limit statistical power.

The non-significant decline observed from 1999-2014 (APC = -0.218, p = 0.288) represents a 15-year period of modest downward trends in bone cancer mortality. Although this trend did not reach statistical significance, its clinical significance becomes apparent when viewed in historical context. This period coincided with the implementation of several important advances in bone cancer management, including the refinement of multimodal treatment protocols combining neoadjuvant chemotherapy with limb-salvage surgery, improved imaging techniques for surgical planning, and enhanced supportive care measures. The gradual nature of this decline may reflect the slow but steady adoption of these evidence-based practices across healthcare systems, with benefits accruing gradually at the population level. The magnitude of the annual percentage change, while modest, suggests that therapeutic innovations during this period may have had meaningful clinical impact that was beginning to translate into population-level mortality reductions. Similarly, the non-significant increase from 2014-2018 (APC = 4.479, p = 0.157) deserves clinical attention despite the lack of statistical significance. The magnitude of this annual percentage change (4.479%) is substantial and suggests a potentially meaningful trend that may not have achieved significance due to the relatively short duration of this segment and the limited number of events in this rare disease. This period coincides with several concerning developments in the healthcare landscape, including the peak of the opioid epidemic, potential disruptions in preventive care and early detection programs, and demographic shifts toward an aging population with higher baseline cancer risk. The clinical significance of this trend is underscored by its consistency across multiple demographic subgroups and its contrast with the preceding period of decline.

The final segment from 2018-2020 (APC = -1.779, p = 0.424) also warrants consideration despite statistical non-significance. While the short duration of this period limits our ability to draw definitive conclusions, particularly given the potential confounding effects of the COVID-19 pandemic in 2020, this trend may represent early signals of improvement. Potential contributing factors could include full implementation of healthcare access expansions, improved coordination of care for complex cancers, or early adoption of emerging therapeutic approaches. The direction of this trend, while not statistically significant, provides a foundation for monitoring future developments and may inform expectations for continued surveillance. When examining demographic subgroups, several non-significant trends also merit clinical consideration. For example, among certain geographic regions and racial/ethnic groups, non-significant trends may reflect emerging disparities or improvements that, while not yet statistically detectable due to sample size constraints, could represent important early signals for targeted public health intervention. These trends may become more apparent with longer follow-up periods or when integrated with other health indicators. The clinical relevance of monitoring these patterns lies in their potential to inform proactive rather than reactive public health responses. It is important to emphasize that our interpretation remains appropriately cautious, as non-significant trends could represent random variation. However, when considered within the broader context of healthcare system evolution, demographic transitions, known therapeutic advances, and documented policy changes, these temporal patterns may provide valuable insights that extend beyond their statistical significance. This nuanced interpretation is particularly important for rare diseases like bone cancer, where the combination of small case numbers and multiple competing factors can obscure meaningful trends that nonetheless have implications for patient care and public health planning.

The critical timing of this mortality trend inflection requires careful analysis to understand potential underlying drivers. This period coincides with the escalation and peak of the U.S. opioid epidemic (30). Beginning around 2010, the opioid crisis placed unprecedented strain on public health systems through multiple pathways that may have indirectly influenced cancer mortality: resource diversion toward overdose prevention and addiction treatment potentially reduced investments in cancer control initiatives; tighter regulations on opioid prescriptions, including those for palliative care in cancer patients, may have compromised pain management quality and accessibility; additionally, opioid overdose deaths themselves contributed to rising overall mortality (31).

Concurrently, the Patient Protection and Affordable Care Act, enacted in 2010, underwent gradual implementation. While the ACA aimed to improve healthcare access and reduce uninsurance rates through Medicaid expansion and health insurance marketplaces, its effects emerged with significant time lags and varied substantially across states (32, 33). Notably, many Southern states with the highest mortality rates identified in this study were precisely those that opted against Medicaid expansion, suggesting the ACA was insufficient to mitigate preexisting health disadvantages in these regions and may have initially failed to prevent further widening of disparities.

As with all large database studies, our research has several important limitations that should be considered when interpreting the results. Most importantly, the use of aggregated data inherently limits causal inference and introduces the possibility of ecological fallacy. As an ecological study, our findings reveal associations at the group level rather than establishing causal relationships at the individual level. The observed racial and geographic disparities may be influenced by unmeasured confounders such as socioeconomic status, educational attainment, healthcare access, insurance status, and behavioral factors within these groups. Individual-level risk factors cannot be directly inferred from population-level associations, and the group-level patterns we observed should not be extrapolated to predict individual outcomes. This ecological design limits our ability to determine whether the demographic and geographic differences in mortality reflect true biological or genetic susceptibilities, or are primarily driven by differential access to care, treatment quality, or other unmeasured social determinants of health.

Second, the absence of detailed clinicopathological information significantly restricts the depth and nuance of our interpretation. The CDC WONDER database relies on death certificates and ICD-10 codes and lacks critical clinical details including: (1) specific histologic subtypes (e.g., osteosarcoma, chondrosarcoma, Ewing sarcoma, undifferentiated pleomorphic sarcoma), which have dramatically different prognoses and treatment responses; (2) tumor staging information (TNM classification, presence of metastases at diagnosis), which is the strongest predictor of survival; (3) detailed treatment modalities (surgical approaches such as limb-salvage versus amputation, specific chemotherapy regimens and dosages, radiation therapy protocols), which directly impact outcomes; (4) tumor characteristics (grade, anatomical location, surgical margin status), which influence prognosis; and (5) treatment response and disease progression markers. This absence of granular clinical data prevents us from determining whether observed mortality trends reflect changes in disease incidence, shifts in histologic distribution, improvements in treatment efficacy for specific subtypes, stage migration effects, or changes in treatment access and quality. For instance, we cannot distinguish whether higher mortality rates in certain demographic groups result from more aggressive tumor biology, later stage at presentation, suboptimal treatment, or other factors. The clinical heterogeneity of bone cancers—ranging from chemotherapy-responsive osteosarcoma in young patients to chemotherapy-resistant chondrosarcoma in elderly patients—means that population-level trends may mask important subtype-specific patterns that would be clinically actionable.

Third, unmeasured confounding factors could significantly affect outcomes: the database does not include individual-level data on socioeconomic status (e.g., education, income, occupation), lifestyle factors (e.g., smoking, obesity), comorbidity indices (e.g., Charlson Comorbidity Index), or environmental exposures, all of which may substantially influence survival outcomes. The aggregated nature of the data prevents us from controlling for these potentially important confounders, limiting our ability to identify the specific mechanisms driving the observed disparities. Additionally, we lack information on healthcare system factors such as access to specialized sarcoma centers, multidisciplinary team care, clinical trial participation, and adherence to evidence-based treatment protocols, all of which are known to significantly impact bone cancer outcomes.

Fourth, the ecological study design may mask important heterogeneity within demographic groups. For example, aggregating all individuals within racial/ethnic categories may obscure important differences in outcomes based on factors such as country of origin, acculturation status, or socioeconomic position within those groups. Similarly, geographic analyses at the state or regional level may not capture local variations in healthcare infrastructure, specialist availability, or population characteristics that could significantly influence mortality patterns. Changes in diagnostic practices, coding accuracy, and death certification quality over time may also contribute to apparent temporal trends that do not reflect true changes in disease incidence or case-fatality rates.

Despite these limitations, our study design is well-suited for its primary objectives: describing population-level mortality trends, identifying high-risk groups and regions, and providing evidence for public health planning and resource allocation. The national representativeness and long-term follow-up provided by CDC WONDER data offer unique advantages for understanding the epidemiologic patterns of rare diseases like bone cancer. However, our findings should be interpreted as hypothesis-generating and require validation through individual-level studies with detailed clinical and sociodemographic data to establish causal mechanisms and inform targeted interventions. Future research should prioritize linkage studies combining mortality databases with clinical registries, multi-institutional clinical database studies, and prospective cohort studies designed to investigate the relationship between sociodemographic factors, clinical characteristics, treatment patterns, and outcomes in bone cancer patients.

In summary, this analysis reveals a concerning public health reality: bone malignant tumor mortality among U.S. adults increased significantly during the first two decades of the 21st century, contrary to expectations of decline. Preexisting health inequities across racial, geographic, and rural-urban dimensions not only persisted but intensified. Most notably, a critical inflection point around 2013 marked an accelerated upward mortality trend across nearly all subgroups, potentially linked to the concurrent opioid crisis and complex healthcare policy interactions. These findings underscore alarming shortcomings—current treatment advances and public health strategies have failed to benefit all patient groups equally and have not effectively reduced health disparities. Urgent intervention is needed through targeted, multi-sector approaches including increased healthcare investment in underserved areas, development of clinical guidelines tailored to vulnerable populations, and policies eliminating healthcare access barriers to reverse rising mortality trends and advance health equity in cancer prevention and control.

## Data Availability

The original contributions presented in the study are included in the article/**Supplementary Material**. Further inquiries can be directed to the corresponding author.

## References

[1] Siegel RL Miller KD Fuchs HE Jemal A . Cancer statistics, 2022. CA: Cancer J Clin. (2022) 72:7–33. doi: 10.3322/caac.21708, PMID: 35020204

[2] Moore DD Luu HH . Osteosarcoma. Cancer Treat Res. (2014) 162:65–92. doi: 10.1007/978-3-319-07323-1_4, PMID: 25070231

[3] Ferguson JL Turner SP . Bone cancer: diagnosis and treatment principles. Am Family Physician. (2018) 98:205–13. 30215968

[4] Isakoff MS Bielack SS Meltzer P Gorlick R . Osteosarcoma: current treatment and a collaborative pathway to success. J Clin Oncol. (2015) 33:3029–35. doi: 10.1200/JCO.2014.59.4895, PMID: 26304877 PMC4979196

[5] Ludwig JA Meyers PA Dirksen U . Ewing’s sarcoma. New Engl J Med. (2021) 384:1476. doi: 10.1056/NEJMc2102423, PMID: 33852792

[6] Gatta G Botta L Rossi S Aareleid T Bielska-Lasota M Clavel J . Childhood cancer survival in Europe 1999-2007: results of EUROCARE-5--a population-based study. Lancet Oncol. (2014) 15:35–47. doi: 10.1016/S1470-2045(13)70548-5, PMID: 24314616

[7] Mirabello L Troisi RJ Savage SA . Osteosarcoma incidence and survival rates from 1973 to 2004: data from the Surveillance, Epidemiology, and End Results Program. Cancer. (2009) 115:1531–43. doi: 10.1002/cncr.24121, PMID: 19197972 PMC2813207

[8] Duchman KR Gao Y Miller BJ . Prognostic factors for survival in patients with high-grade osteosarcoma using the Surveillance, Epidemiology, and End Results (SEER) Program database. Cancer Epidemiol. (2015) 39:593–9. doi: 10.1016/j.canep.2015.05.001, PMID: 26002013

[9] Esiashvili N Goodman M Marcus RB Jr. Changes in incidence and survival of Ewing sarcoma patients over the past 3 decades: Surveillance Epidemiology and End Results data. J Pediatr Hematol/Oncol. (2008) 30:425–30. doi: 10.1097/MPH.0b013e31816e22f3, PMID: 18525458

[10] Hu X Fujiwara T Houdek MT Chen L Huang W Sun Z . Impact of racial disparities and insurance status in patients with bone sarcomas in the USA: a population-based cohort study. Bone Joint Res. (2022) 11:278–91. doi: 10.1302/2046-3758.115.BJR-2021-0258.R2, PMID: 35549518 PMC9130676

[11] Jacobs AJ Lindholm EB Levy CF Fish JD Glick RD . Racial and ethnic disparities in treatment and survival of pediatric sarcoma. J Surg Res. (2017) 219:43–9. doi: 10.1016/j.jss.2017.05.031, PMID: 29078908

[12] Bhagwan R Nabi R Rath S Chaudhry SAA Meghwar S Rathi D . Trends in sudden cardiac death related mortality in adults in the United States: A CDC WONDER database analysis, 1999-2020. Clin Cardiol. (2025) 48:e70180. doi: 10.1002/clc.70180, PMID: 40662462 PMC12261031

[13] Kim HJ Fay MP Feuer EJ Midthune DN . Permutation tests for joinpoint regression with applications to cancer rates. Stat Med. (2000) 19:335–51. doi: 10.1002/(SICI)1097-0258(20000215)19:3<335::AID-SIM336>3.0.CO;2-Z 10649300

[14] van Praag Veroniek VM Rueten-Budde AJ Ho V Dijkstra PDS Fiocco M van de Sande MAJ . Incidence, outcomes and prognostic factors during 25 years of treatment of chondrosarcomas. Surg Oncol. (2018) 27:402–8. doi: 10.1016/j.suronc.2018.05.009, PMID: 30217294

[15] Bergh P Gunterberg B Meis-Kindblom JM Kindblom LG . Prognostic factors and outcome of pelvic, sacral, and spinal chondrosarcomas: a center-based study of 69 cases. Cancer. (2001) 91:1201–12. doi: 10.1002/1097-0142(20010401)91:7<1201::AID-CNCR1120>3.0.CO;2-W 11283918

[16] Bacci G Longhi A Briccoli A Bertoni F Versari M Picci P . The role of surgical margins in treatment of Ewing’s sarcoma family tumors: experience of a single institution with 512 patients treated with adjuvant and neoadjuvant chemotherapy. Int J Radiat Oncol Biol Physics. (2006) 65:766–72. doi: 10.1016/j.ijrobp.2006.01.019, PMID: 16626886

[17] Smith-Bindman R Miglioretti DL Johnson E Lee C Feigelson HS Flynn M . Use of diagnostic imaging studies and associated radiation exposure for patients enrolled in large integrated health care systems, 1996-2010. Jama. (2012) 307:2400–9. doi: 10.1001/jama.2012.5960, PMID: 22692172 PMC3859870

[18] Lagmay JP Krailo MD Dang H Kim A Hawkins DS Beaty O 3rd . Outcome of patients with recurrent osteosarcoma enrolled in seven phase II trials through children’s cancer group, pediatric oncology group, and children’s oncology group: learning from the past to move forward. J Clin Oncol. (2016) 34:3031–8. doi: 10.1200/JCO.2015.65.5381, PMID: 27400942 PMC5012712

[19] Chawla S Blay JY Rutkowski P Le Cesne A Reichardt P Gelderblom H . Denosumab in patients with giant-cell tumour of bone: a multicentre, open-label, phase 2 study. Lancet Oncol. (2019) 20:1719–29. doi: 10.1016/S1470-2045(19)30663-1, PMID: 31704134

[20] Tawbi HA Burgess M Bolejack V Van Tine BA Schuetze SM Hu J . Pembrolizumab in advanced soft-tissue sarcoma and bone sarcoma (SARC028): a multicentre, two-cohort, single-arm, open-label, phase 2 trial. Lancet Oncol. (2017) 18:1493–501. doi: 10.1016/S1470-2045(17)30624-1, PMID: 28988646 PMC7939029

[21] Gill J Ahluwalia MK Geller D Gorlick R . New targets and approaches in osteosarcoma. Pharmacol Ther. (2013) 137:89–99. doi: 10.1016/j.pharmthera.2012.09.003, PMID: 22983152

[22] Blay JY De Pinieux G Gouin F . Ewing’s sarcoma. New Engl J Med. (2021) 384:1477. doi: 10.1056/NEJMc2102423, PMID: 33852793

[23] Iqbal J Ginsburg O Rochon PA Sun P Narod SA . Differences in breast cancer stage at diagnosis and cancer-specific survival by race and ethnicity in the United States. Jama. (2015) 313:165–73. doi: 10.1001/jama.2014.17322, PMID: 25585328

[24] Esnaola NF Ford ME . Racial differences and disparities in cancer care and outcomes: where’s the rub? Surg Oncol Clinics North America. (2012) 21:417–37, viii. doi: 10.1016/j.soc.2012.03.012, PMID: 22583991 PMC4180671

[25] Rahib L Smith BD Aizenberg R Rosenzweig AB Fleshman JM Matrisian LM . Projecting cancer incidence and deaths to 2030: the unexpected burden of thyroid, liver, and pancreas cancers in the United States. Cancer Res. (2014) 74:2913–21. doi: 10.1158/0008-5472.CAN-14-0155, PMID: 24840647

[26] Moy B Polite BN Halpern MT Stranne SK Winer EP Wollins DS . American Society of Clinical Oncology policy statement: opportunities in the patient protection and affordable care act to reduce cancer care disparities. J Clin Oncol. (2011) 29:3816–24. doi: 10.1200/JCO.2011.35.8903, PMID: 21810680

[27] Blake KD Moss JL Gaysynsky A Srinivasan S Croyle RT . Making the case for investment in rural cancer control: an analysis of rural cancer incidence, mortality, and funding trends. Cancer Epidemiol Biomarkers Prev. (2017) 26:992–7. doi: 10.1158/1055-9965.EPI-17-0092, PMID: 28600296 PMC5500425

[28] Douthit N Kiv S Dwolatzky T Biswas S . Exposing some important barriers to health care access in the rural USA. Public Health. (2015) 129:611–20. doi: 10.1016/j.puhe.2015.04.001, PMID: 26025176

[29] Semprini J Gadag K Williams G Muldrow A Zahnd WE . Rural-urban cancer incidence and trends in the United States, 2000 to 2019. Cancer Epidemiol Biomarkers Prev. (2024) 33:1012–22. doi: 10.1158/1055-9965.EPI-24-0072, PMID: 38801414

[30] Ruhm CJ . Drivers of the fatal drug epidemic. J Health Econ. (2019) 64:25–42. doi: 10.1016/j.jhealeco.2019.01.001, PMID: 30784811

[31] Jayawardana S Forman R Johnston-Webber C Campbell A Berterame S de Joncheere C . Global consumption of prescription opioid analgesics between 2009-2019: a country-level observational study. EClinicalMedicine. (2021) 42:101198. doi: 10.1016/j.eclinm.2021.101198, PMID: 34820610 PMC8599097

[32] Sommers BD Gunja MZ Finegold K Musco T . Changes in self-reported insurance coverage, access to care, and health under the affordable care act. Jama. (2015) 314:366–74. doi: 10.1001/jama.2015.8421, PMID: 26219054

[33] Han X Robin Yabroff K Guy GP Jr. Zheng Z Jemal A . Has recommended preventive service use increased after elimination of cost-sharing as part of the Affordable Care Act in the United States? Prev Med. (2015) 78:85–91. doi: 10.1016/j.ypmed.2015.07.012, PMID: 26209914 PMC4589867

