# Supplementary material for: Trends and disparities in bone cancer mortality among US adults from 1999 to 2020: a joinpoint regression analysis based on the CDC WONDER database
Source: Front Oncol. 2026 Mar 5;16:1718354. doi: 10.3389/fonc.2026.1718354 (PMC12999440; doi:10.3389/fonc.2026.1718354)
Supplement: Supplementary file 1 [file DataSheet1.doc]

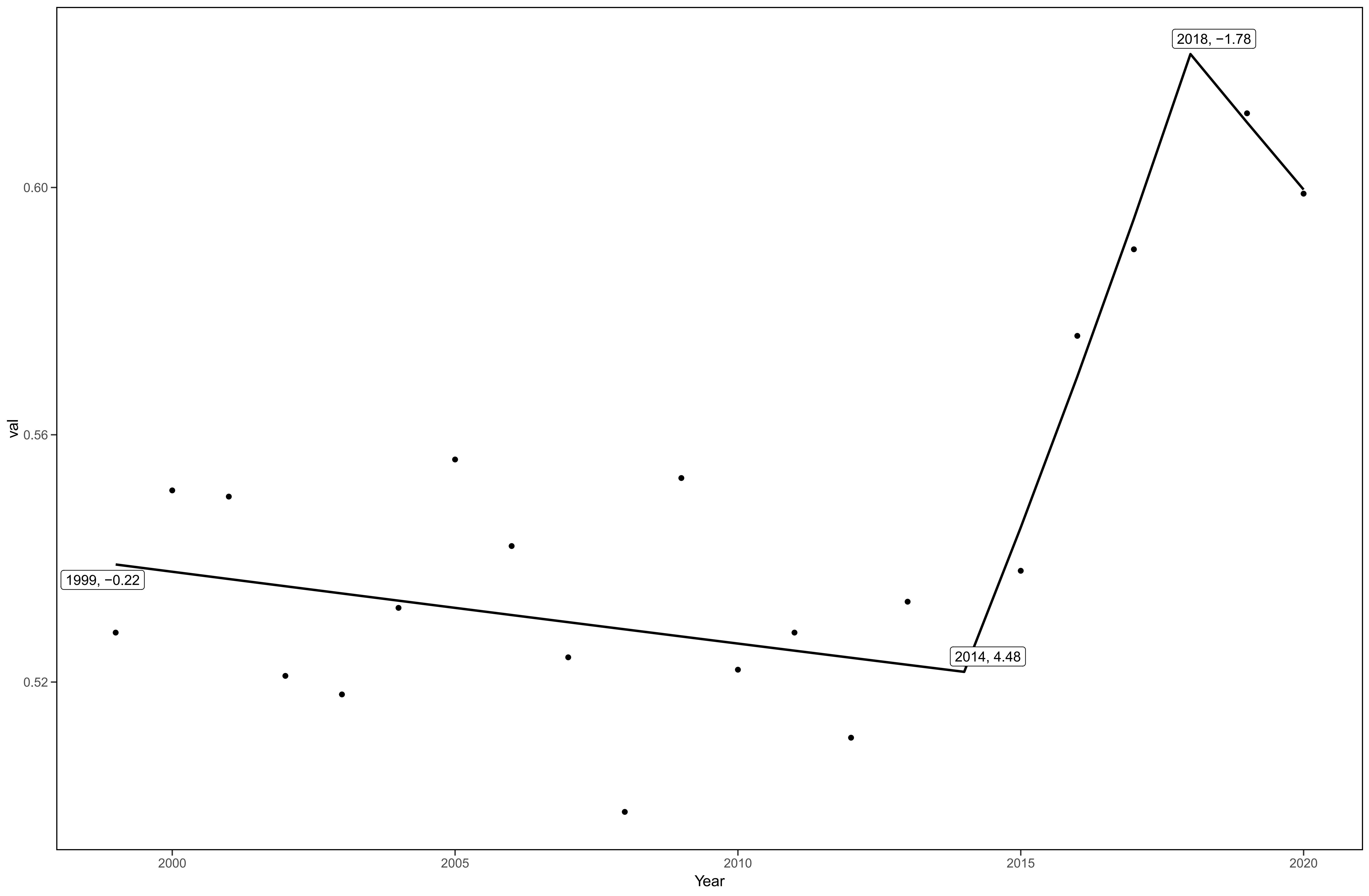


**Supplementary Figure S1.Age-Adjusted Mortality Rates Annual Percentage Change (APC) from Bone cancer Deaths in the US Annual Trends(1999-2020).**

**
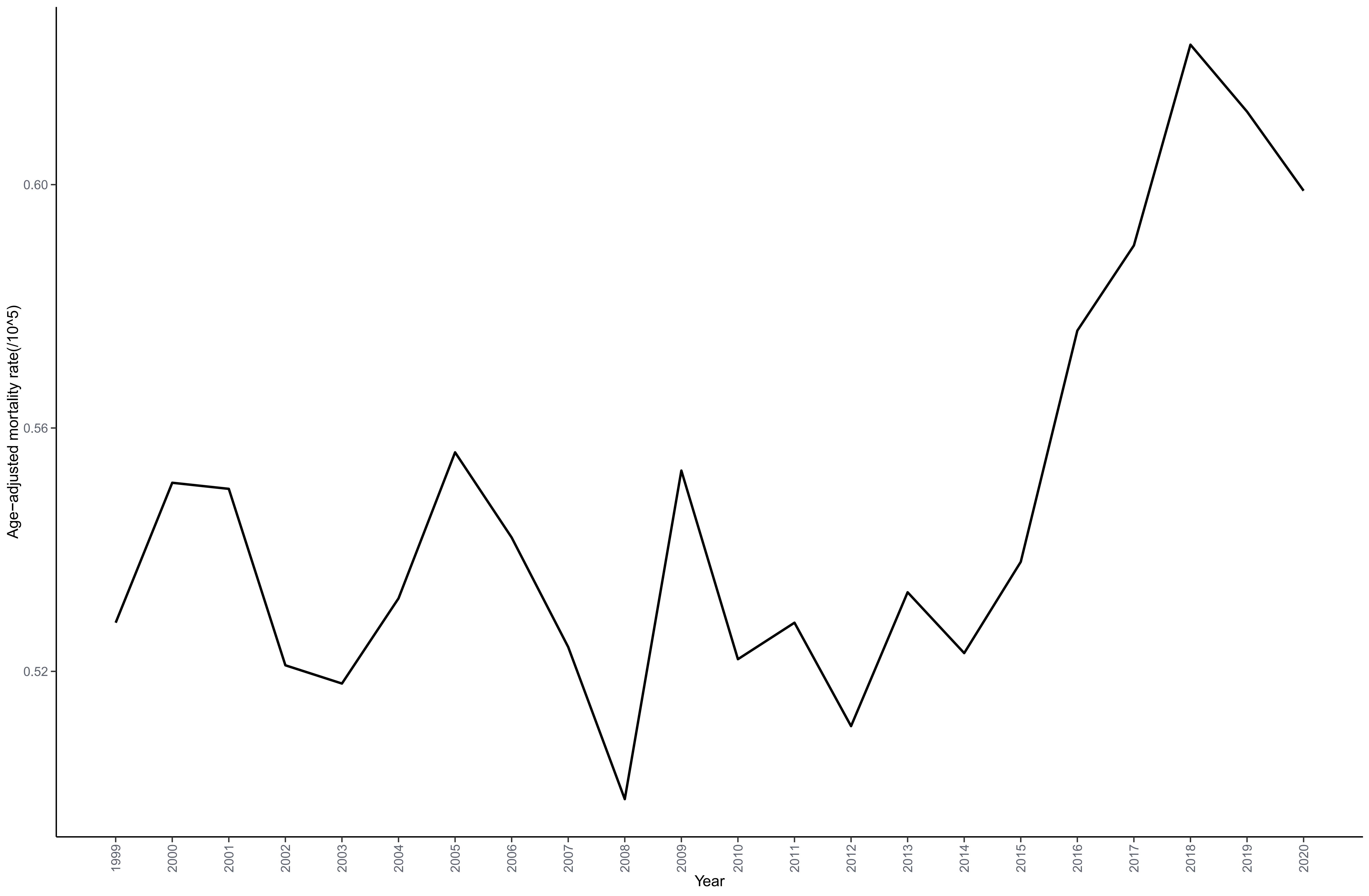
**

**Supplementary Figure S2.Trends in age-adjusted mortality rate for bone cancer-related mortality in the United States(1999-2020).**


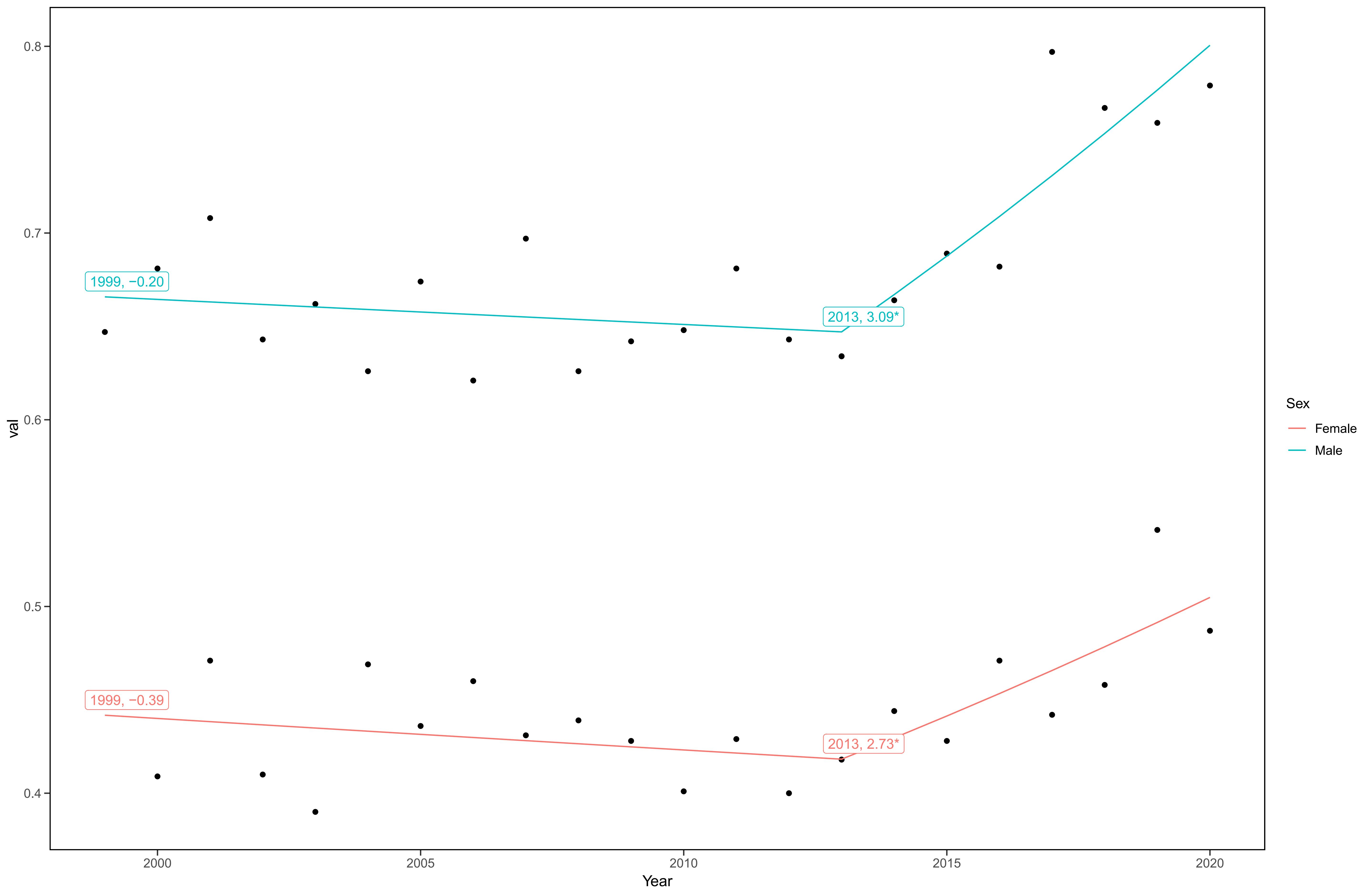


**Supplementary Figure S3.Age-Adjusted Mortality Rates Annual Percentage Change (APC) from Sudden Cardiac Deaths in the US by Gender (1999-2020).**


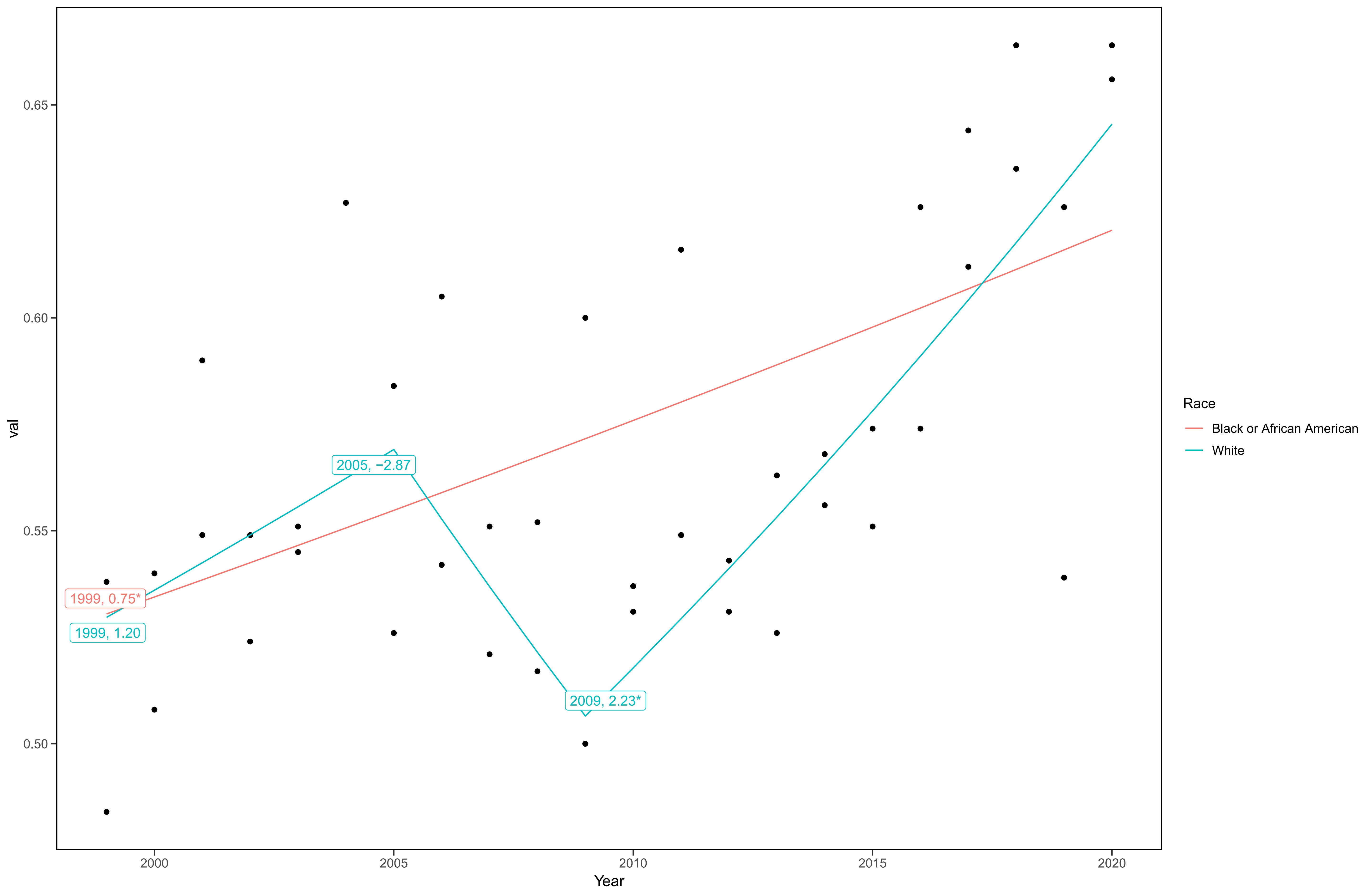


**Supplementary Figure S4.Age-Adjusted Mortality Rates Annual Percentage Change (APC) from Sudden Cardiac Deaths in the US by Race (1999-2020).**

**
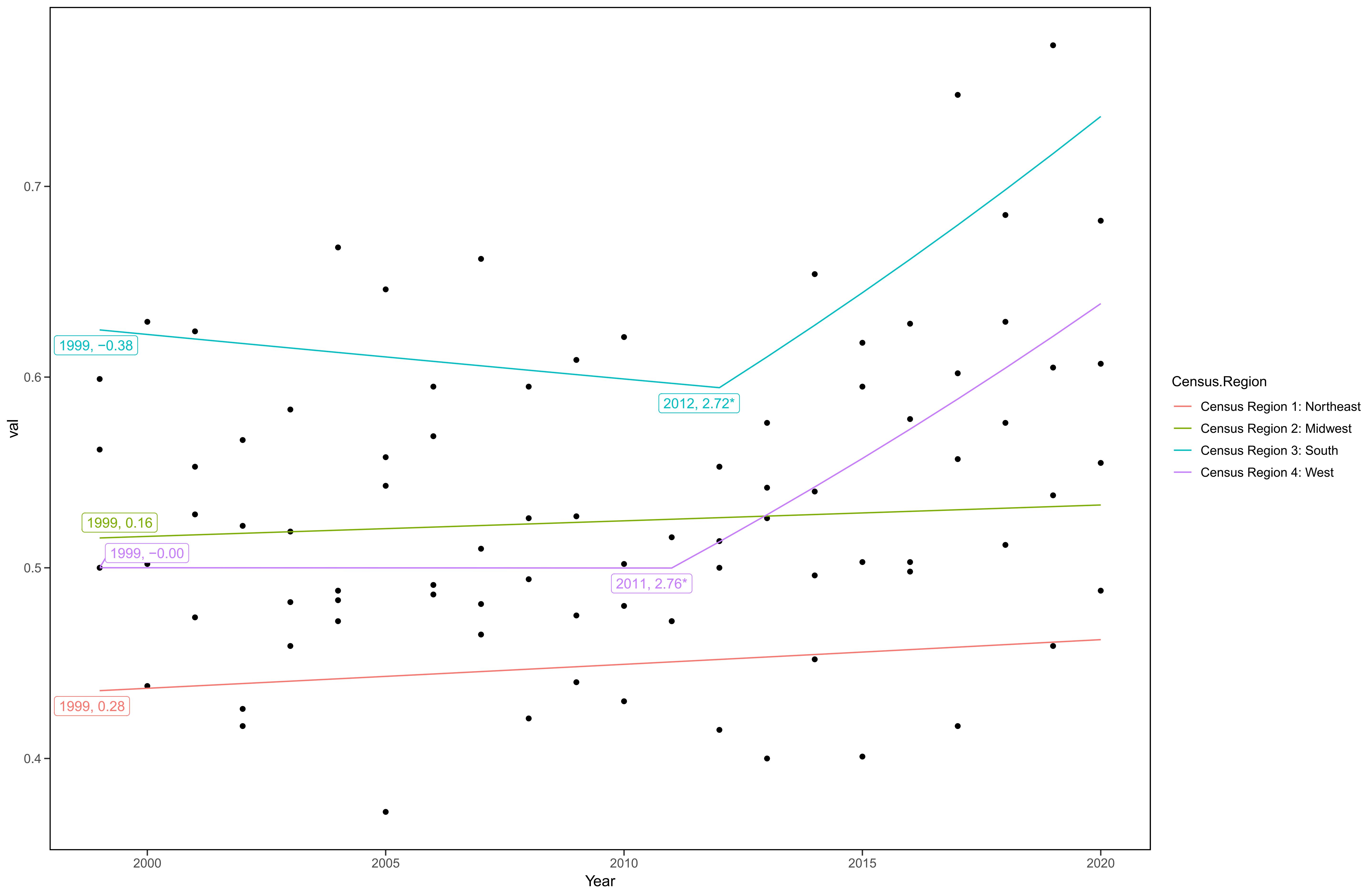
**

**Supplementary Figure S5.Age-Adjusted Mortality Rates Annual Percentage Change (APC) from Sudden Cardiac Deaths in the US by Region (1999-2020).**


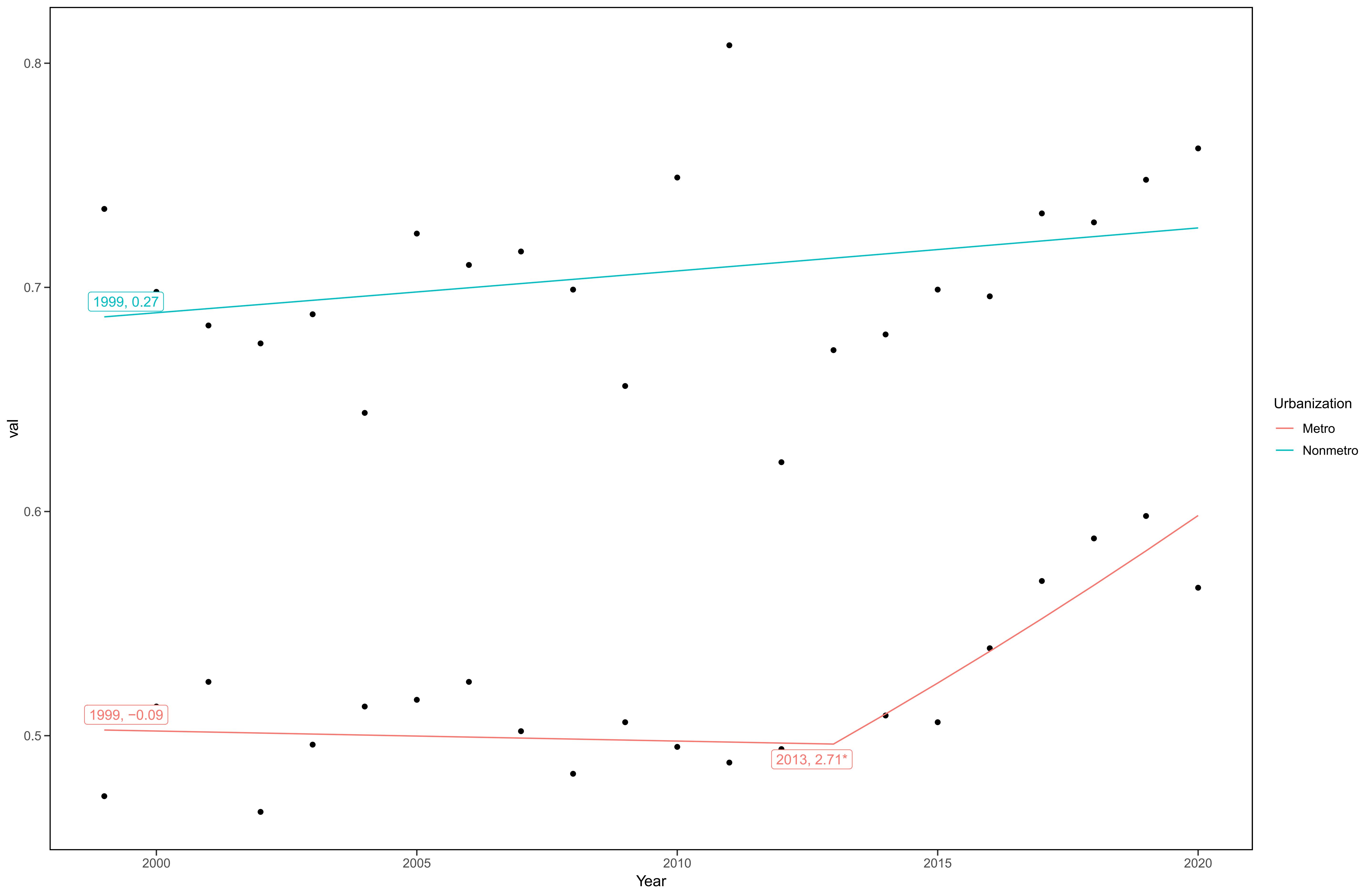


**Supplementary Figure S6.Age-Adjusted Mortality Rates Annual Percentage Change (APC) from Sudden Cardiac Deaths in the US by Metropolitan Status (1999-2020).**

**Supplementary Table S1. Bone cancer-related mortality in US adults overall and by sex and ethnic stratification from 1999 to 2020.**

|  | **Overall** | **Female** | **Male** | **Hispanic or Latino** | **Black or African American** | **White people** | **Population** |
| --- | --- | --- | --- | --- | --- | --- | --- |
| **1999** | 940 | 437 | 503 | 17 | 88 | 835 | 180408769 |
| **2000** | 954 | 427 | 527 | 17 | 87 | 850 | 181984640 |
| **2001** | 1015 | 481 | 534 | 25 | 111 | 879 | 184305128 |
| **2002** | 934 | 418 | 516 | 19 | 104 | 811 | 186208028 |
| **2003** | 990 | 441 | 549 | 24 | 101 | 865 | 188090429 |
| **2004** | 1031 | 488 | 543 | 23 | 122 | 886 | 190205384 |
| **2005** | 1074 | 480 | 594 | 24 | 107 | 943 | 192551384 |
| **2006** | 1071 | 500 | 571 | 33 | 121 | 917 | 195019359 |
| **2007** | 1088 | 470 | 618 | 27 | 101 | 960 | 197403777 |
| **2008** | 1072 | 484 | 588 | 38 | 118 | 916 | 199795090 |
| **2009** | 1091 | 486 | 605 | 37 | 135 | 919 | 202107016 |
| **2010** | 1101 | 470 | 631 | 33 | 123 | 945 | 203891983 |
| **2011** | 1141 | 507 | 634 | 27 | 133 | 981 | 206592936 |
| **2012** | 1119 | 482 | 637 | 34 | 125 | 960 | 208826037 |
| **2013** | 1180 | 522 | 658 | 36 | 128 | 1016 | 211085314 |
| **2014** | 1262 | 567 | 695 | 47 | 145 | 1070 | 213809280 |
| **2015** | 1275 | 529 | 746 | 41 | 139 | 1095 | 216553817 |
| **2016** | 1351 | 607 | 744 | 55 | 154 | 1142 | 218641417 |
| **2017** | 1475 | 612 | 863 | 54 | 174 | 1247 | 221447331 |
| **2018** | 1542 | 671 | 871 | 58 | 177 | 1307 | 223311190 |
| **2019** | 1566 | 723 | 843 | 65 | 158 | 1343 | 224981167 |
| **2020** | 1587 | 694 | 893 | 58 | 190 | 1339 | 226635013 |
| **Total** | 25859 | 11496 | 14363 | 792 | 2841 | 22226 | 4473854489 |

**Supplementary Table S2.Age-Adjusted Mortality Rates (AAMR) from Bone cancer Deaths in the US by Gender (1999-2020).**

| **Year** | **Overall** | **Female** | **Male** |
| --- | --- | --- | --- |
| **1999** | 0.528(0.495-0.562) | 0.452(0.410-0.495) | 0.647(0.589-0.705) |
| **2000** | 0.551(0.516-0.586) | 0.409(0.370-0.448) | 0.681(0.622-0.740) |
| **2001** | 0.550(0.516-0.584) | 0.471(0.428-0.514) | 0.708(0.647-0.768) |
| **2002** | 0.521(0.488-0.555) | 0.410(0.370-0.450) | 0.643(0.586-0.699) |
| **2003** | 0.518(0.486-0.550) | 0.39(0.353-0.426) | 0.662(0.606-0.719) |
| **2004** | 0.532(0.500-0.565) | 0.469(0.427-0.511) | 0.626(0.572-0.680) |
| **2005** | 0.556(0.522-0.589) | 0.436(0.396-0.475) | 0.674(0.619-0.729) |
| **2006** | 0.542(0.510-0.575) | 0.460(0.419-0.501) | 0.621(0.569-0.673) |
| **2007** | 0.524(0.492-0.555) | 0.431(0.392-0.471) | 0.697(0.641-0.753) |
| **2008** | 0.499(0.469-0.530) | 0.439(0.399-0.479) | 0.626(0.574-0.677) |
| **2009** | 0.553(0.520-0.586) | 0.428(0.389-0.467) | 0.642(0.590-0.694) |
| **2010** | 0.522(0.490-0.553) | 0.401(0.364-0.439) | 0.648(0.596-0.700) |
| **2011** | 0.528(0.497-0.559) | 0.429(0.390-0.468) | 0.681(0.627-0.735) |
| **2012** | 0.511(0.481-0.542) | 0.400(0.363-0.437) | 0.643(0.593-0.694) |
| **2013** | 0.533(0.502-0.564) | 0.418(0.381-0.454) | 0.634(0.585-0.684) |
| **2014** | 0.523(0.493-0.552) | 0.444(0.407-0.481) | 0.664(0.613-0.714) |
| **2015** | 0.538(0.508-0.568) | 0.428(0.390-0.467) | 0.689(0.638-0.739) |
| **2016** | 0.576(0.545-0.607) | 0.471(0.432-0.510) | 0.682(0.632-0.732) |
| **2017** | 0.590(0.559-0.621) | 0.442(0.406-0.477) | 0.797(0.743-0.852) |
| **2018** | 0.623(0.591-0.654) | 0.458(0.423-0.493) | 0.767(0.715-0.819) |
| **2019** | 0.612(0.581-0.643) | 0.541(0.500-0.581) | 0.759(0.706-0.811) |
| **2020** | 0.599(0.569-0.628) | 0.487(0.449-0.524) | 0.779(0.726-0.831) |
| **Total** | 0.538(0.531-0.544) | 0.446(0.437-0.454) | 0.712(0.700-0.723) |

**Age-Adjusted Rate (95% CI)**

**Supplementary Table S3.Age-Adjusted Mortality Rates (AAMR) from Bone cancer Deaths in the US by Race (1999-2020).**

| **Year** | **Hispanic or Latino** | **Black or African American** | **White people** |
| --- | --- | --- | --- |
| **1999** | **-** | 0.484(0.385-0.599) | 0.538(0.501-0.574) |
| **2000** | - | 0.508(0.404-0.631) | 0.540(0.504-0.576) |
| **2001** | 0.372(0.236-0.558) | 0.590(0.476-0.703) | 0.549(0.513-0.586) |
| **2002** | - | 0.549(0.439-0.680) | 0.524(0.487-0.560) |
| **2003** | 0.302(0.184-0.466) | 0.551(0.442-0.661) | 0.545(0.509-0.582) |
| **2004** | 0.233(0.142-0.360) | 0.627(0.512-0.741) | 0.566(0.528-0.603) |
| **2005** | 0.251(0.157-0.380) | 0.526(0.422-0.629) | 0.584(0.546-0.621) |
| **2006** | 0.380(0.256-0.542) | 0.605(0.494-0.716) | 0.542(0.507-0.578) |
| **2007** | 0.301(0.195-0.445) | 0.521(0.416-0.620) | 0.551(0.516-0.587) |
| **2008** | 0.339(0.232-0.478) | 0.552(0.450-0.650) | 0.517(0.483-0.550) |
| **2009** | 0.346(0.238-0.486) | 0.600(0.495-0.705) | 0.500(0.467-0.533) |
| **2010** | 0.295(0.198-0.424) | 0.537(0.439-0.634) | 0.531(0.497-0.565) |
| **2011** | 0.249(0.161-0.368) | 0.616(0.5508-0.724) | 0.549(0.514-0.584) |
| **2012** | 0.283(0.191-0.404) | 0.543(0.445-0.641) | 0.531(0.497-0.566) |
| **2013** | 0.254(0.175-0.357) | 0.526(0.432-0.619) | 0.563(0.528-0.598) |
| **2014** | 0.370(0.269-0.497) | 0.568(0.473-0.664) | 0.556(0.522-0.590) |
| **2015** | 0.281(0.199-0.385) | 0.574(0.476-0.673) | 0.551(0.518-0.584) |
| **2016** | 0.399(0.299-0.523) | 0.626(0.240-0.728) | 0.574(0.540-0.608) |
| **2017** | 0.362(0.271-0.475) | 0.644(0.545-0.742) | 0.612(0.577-0.646) |
| **2018** | 0.368(0.278-0.478) | 0.664(0.562-0.765) | 0.635(0.600-0.670) |
| **2019** | 0.406(0.312-0.519) | 0.539(0.452-0.625) | 0.626(0.592-0.660) |
| **2020** | 0.358(0.270-0.464) | 0.664(0.567-0.762) | 0.656(0.620-0.698) |
| **Total** | 0.335(0.311-0.359) | 0.584(0.562-0.606) | 0.561(0.554-0.568) |

**Age-Adjusted Rate (95% CI)**

**Supplementary Table S4.Age-Adjusted Mortality Rates (AAMR) from Bone cancer Deaths in the US by Urban-rural classification (1999-2020).**

| **Year** | **Metropolitan** | **Non-Metropolitan** |
| --- | --- | --- |
| **1999** | 0.473(0.438-0.508) | 0.735(0.640-0.830) |
| **2000** | 0.513(0.476-0.551) | 0.698(0.605-0.790) |
| **2001** | 0.524(0.487-0.560) | 0.683(0.592-0.774) |
| **2002** | 0.466(0.431-0.500) | 0.675(0.586-0.764) |
| **2003** | 0.496(0.461-0.531) | 0.688(0.599-0.778) |
| **2004** | 0.513(0.477-0.548) | 0.644(0.558-0.729) |
| **2005** | 0.516(0.480-0.551) | 0.724(0.630-0.817) |
| **2006** | 0.524(0.488-0.559) | 0.710(0.619-0.802) |
| **2007** | 0.502(0.468-0.360) | 0.716(0.624-0.808) |
| **2008** | 0.483(0.450-0.516) | 0.699(0.610-0.787) |
| **2009** | 0.506(0.472-0.540) | 0.656(0.568-0.744) |
| **2010** | 0.495(0.461-0.529) | 0.749(0.655-0.844) |
| **2011** | 0.488(0.455-0.521) | 0.808(0.710-0.906) |
| **2012** | 0.494(0.461-0.527) | 0.622(0.538-0.706) |
| **2013** | 0.488(0.456-0.520) | 0.672(0.587-0.758) |
| **2014** | 0.509(0.477-0.541) | 0.679(0.592-0.766) |
| **2015** | 0.506(0.475-0.538) | 0.699(0.613-0.785) |
| **2016** | 0.539(0.507-0.572) | 0.696(0.610-0.782) |
| **2017** | 0.569(0.536-0.603) | 0.733(0.644-0.822) |
| **2018** | 0.588(0.555-0.621) | 0.729(0.644-0.814) |
| **2019** | 0.598(0.565-0.632) | 0.748(0.660-0.835) |
| **2020** | 0.566(0.535-0.598) | 0.762(0.674-0.849) |
| **Total** | 0.511(0.504-0.518) | 0.698(0.679-0.717) |

**Age-Adjusted Rate (95% CI)**

**Supplementary Table S5.Age-Adjusted Mortality Rates (AAMR) from Bone cancer Deaths in the US by Census region (1999-2020).**

| **Census Region** | **Year** | **Age-Adjusted Rate with 95% CI** |
| --- | --- | --- |
| **Census Region 1: Northeast** | 1999 | 0.425(0.357-0.493) |
| **Census Region 1: Northeast** | 2000 | 0.438(0.370-0.507) |
| **Census Region 1: Northeast** | 2001 | 0.474(0.403-0.546) |
| **Census Region 1: Northeast** | 2002 | 0.417(0.351-0.482) |
| **Census Region 1: Northeast** | 2003 | 0.459(0.391-0.526) |
| **Census Region 1: Northeast** | 2004 | 0.483(0.413-0.553) |
| **Census Region 1: Northeast** | 2005 | 0.372(0.311-0.434) |
| **Census Region 1: Northeast** | 2006 | 0.486(0.415-0.556) |
| **Census Region 1: Northeast** | 2007 | 0.465(0.398-0.531) |
| **Census Region 1: Northeast** | 2008 | 0.421(0.356-0.486) |
| **Census Region 1: Northeast** | 2009 | 0.440(0.374-0.506) |
| **Census Region 1: Northeast** | 2010 | 0.430(0.365-0.495) |
| **Census Region 1: Northeast** | 2011 | 0.491(0.421-0.561) |
| **Census Region 1: Northeast** | 2012 | 0.415(0.355-0.476) |
| **Census Region 1: Northeast** | 2013 | 0.400(0.339-0.460) |
| **Census Region 1: Northeast** | 2014 | 0.452(0.387-0.517) |
| **Census Region 1: Northeast** | 2015 | 0.401(0.338-0.464) |
| **Census Region 1: Northeast** | 2016 | 0.503(0.434-0.573) |
| **Census Region 1: Northeast** | 2017 | 0.417(0.356-0.478) |
| **Census Region 1: Northeast** | 2018 | 0.512(0.444-0.580) |
| **Census Region 1: Northeast** | 2019 | 0.459(0.396-0.523) |
| **Census Region 1: Northeast** | 2020 | 0.488(0.423-0.552) |
| **Census Region 1: Northeast** | Total | 0.444(0.430-0.458) |
| **Census Region 2: Midwest** | 1999 | 0.562(0.490-0.635) |
| **Census Region 2: Midwest** | 2000 | 0.504(0.435-0.573) |
| **Census Region 2: Midwest** | 2001 | 0.528(0.459-0.597) |
| **Census Region 2: Midwest** | 2002 | 0.522(0.453-0.591) |
| **Census Region 2: Midwest** | 2003 | 0.519(0.452-0.586) |
| **Census Region 2: Midwest** | 2004 | 0.472(0.405-0.538) |
| **Census Region 2: Midwest** | 2005 | 0.543(0.474-0.611) |
| **Census Region 2: Midwest** | 2006 | 0.569(0.498-0.641) |
| **Census Region 2: Midwest** | 2007 | 0.510(0.443-0.578) |
| **Census Region 2: Midwest** | 2008 | 0.526(0.458-0.594) |
| **Census Region 2: Midwest** | 2009 | 0.475(0.411-0.539) |
| **Census Region 2: Midwest** | 2010 | 0.502(0.439-0.566) |
| **Census Region 2: Midwest** | 2011 | 0.516(0.450-0.583) |
| **Census Region 2: Midwest** | 2012 | 0.500(0.434-0.566) |
| **Census Region 2: Midwest** | 2013 | 0.542(0.474-0.611) |
| **Census Region 2: Midwest** | 2014 | 0.496(0.434-0.558) |
| **Census Region 2: Midwest** | 2015 | 0.503(0.439-0.568) |
| **Census Region 2: Midwest** | 2016 | 0.498(0.435-0.560) |
| **Census Region 2: Midwest** | 2017 | 0.557(0.492-0.621) |
| **Census Region 2: Midwest** | 2018 | 0.576(0.508-0.643) |
| **Census Region 2: Midwest** | 2019 | 0.538(0.475-0.601) |
| **Census Region 2: Midwest** | 2020 | 0.555(0.491-0.620) |
| **Census Region 2: Midwest** | Total | 0.614(0.602-0.626) |
| **Census Region 3: South** | 1999 | 0.599(0.538-0.660) |
| **Census Region 3: South** | 2000 | 0.629(0.567-0.690) |
| **Census Region 3: South** | 2001 | 0.624(0.563-0.685) |
| **Census Region 3: South** | 2002 | 0.567(0.510-0.624) |
| **Census Region 3: South** | 2003 | 0.583(0.525-0.642) |
| **Census Region 3: South** | 2004 | 0.668(0.606-0.730) |
| **Census Region 3: South** | 2005 | 0.646(0.586-0.706) |
| **Census Region 3: South** | 2006 | 0.595(0.538-0.652) |
| **Census Region 3: South** | 2007 | 0.662(0.602-0.722) |
| **Census Region 3: South** | 2008 | 0.595(0.540-0.651) |
| **Census Region 3: South** | 2009 | 0.609(0.553-0.665) |
| **Census Region 3: South** | 2010 | 0.621(0.564-0.678) |
| **Census Region 3: South** | 2011 | 0.586(0.532-0.640) |
| **Census Region 3: South** | 2012 | 0.553(0.501-0.605) |
| **Census Region 3: South** | 2013 | 0.576(0.524-0.629) |
| **Census Region 3: South** | 2014 | 0.654(0.599-0.710) |
| **Census Region 3: South** | 2015 | 0.618(0.565-0.670) |
| **Census Region 3: South** | 2016 | 0.628(0.575-0.681) |
| **Census Region 3: South** | 2017 | 0.748(0.690-0.806) |
| **Census Region 3: South** | 2018 | 0.685(0.631-0.739) |
| **Census Region 3: South** | 2019 | 0.774(0.716-0.833) |
| **Census Region 3: South** | 2020 | 0.682(0.630-0.733) |
| **Census Region 3: South** | Total | 0.531(0.517-0.545) |
| **Census Region 4: West** | 1999 | 0.500(0.427-0.574) |
| **Census Region 4: West** | 2000 | 0.502(0.430-0.575) |
| **Census Region 4: West** | 2001 | 0.553(0.478-0.627) |
| **Census Region 4: West** | 2002 | 0.426(0.362-0.490) |
| **Census Region 4: West** | 2003 | 0.482(0.414-0.550) |
| **Census Region 4: West** | 2004 | 0.488(0.420-0.556) |
| **Census Region 4: West** | 2005 | 0.558(0.486-0.630) |
| **Census Region 4: West** | 2006 | 0.491(0.425-0.557) |
| **Census Region 4: West** | 2007 | 0.481(0.414-0.548) |
| **Census Region 4: West** | 2008 | 0.494(0.428-0.559) |
| **Census Region 4: West** | 2009 | 0.527(0.460-0.595) |
| **Census Region 4: West** | 2010 | 0.480(0.415-0.544) |
| **Census Region 4: West** | 2011 | 0.472(0.411-0.534) |
| **Census Region 4: West** | 2012 | 0.514(0.451-0.577) |
| **Census Region 4: West** | 2013 | 0.526(0.463-0.590) |
| **Census Region 4: West** | 2014 | 0.540(0.475-0.605) |
| **Census Region 4: West** | 2015 | 0.595(0.528-0.662) |
| **Census Region 4: West** | 2016 | 0.578(0.513-0.643) |
| **Census Region 4: West** | 2017 | 0.602(0.536-0.668) |
| **Census Region 4: West** | 2018 | 0.629(0.563-0.695) |
| **Census Region 4: West** | 2019 | 0.605(0.541-0.669) |
| **Census Region 4: West** | 2020 | 0.607(0.543-0.671) |
| **Census Region 4: West** | Total | 0.538(0.531-0.544) |

**Supplementary Table S6.Age-Adjusted Mortality Rates (AAMR) from Bone cancer Deaths in the US by State (1999-2020).**

| **State** | **Age-Adjusted Rate with 95% CI** |
| --- | --- |
| **Mississippi** | 1.535(1.419-1.652) |
| **Arkansas** | 1.142(1.043-0.242) |
| **Louisiana** | 0.950(0.875-1.026) |
| **Alabama** | 0.897(0.828-0.966) |
| **Oklahoma** | 0.776(0.702-0.850) |
| **Kentucky** | 0.715(0.650-0.781) |
| **South Carolina** | 0.653(0.592-0.714) |
| **Texas** | 0.625(0.598-0.653) |
| **Kansas** | 0.589(0.514-0.664) |
| **West Virginia** | 0.589(0.503-0.675) |
| **South Dakota** | 0.578(0.453-0.726) |
| **Georgia** | 0.576(0.534-0.619) |
| **Tennessee** | 0.575(0.527-0.623) |
| **Missouri** | 0.574(0.525-0.523) |
| **Wyoming** | 0.569(0.415-0.761) |
| **Michigan** | 0.568(0.530-0.605) |
| **Nevada** | 0.566(0.489-0.643) |
| **New Mexico** | 0.566(0.483-0.648) |
| **Arizona** | 0.564(0.517-0.612) |
| **Utah** | 0.549(0.465-0.632) |
| **Idaho** | 0.545(0.443-0.647) |
| **Nebraska** | 0.541(0.455-0.627) |
| **California** | 0.533(0.513-0.553) |
| **Indiana** | 0.532(0.487-0.578) |
| **Illinois** | 0.525(0.493-0.558) |
| **Montana** | 0.523(0.416-0.649) |
| **Washington** | 0.514(0.469-0.559) |
| **Maryland** | 0.513(0.463-0.562) |
| **Florida** | 0.504(0.480-0.529) |
| **Iowa** | 0.504(0.441-0.567) |
| **North Carolina** | 0.496(0.459-0.533) |
| **Oregon** | 0.488(0.433-0.544) |
| **Pennsylvania** | 0.486(0.456-0.517) |
| **Minnesota** | 0.482(0.432-0.531) |
| **Maine** | 0.479(0.386-0.571) |
| **Colorado** | 0.476(0.425-0.527) |
| **Ohio** | 0.476(0.444-0.509) |
| **Delaware** | 0.466(0.357-0.597) |
| **Rhode Island** | 0.464(0.365-0.581) |
| **Wisconsin** | 0.459(0.415-0.503) |
| **Vermont** | 0.449(0.325-0.605) |
| **Massachusetts** | 0.448(0.406-0.490) |
| **Virginia** | 0.437(0.399-0.476) |
| **New York** | 0.436(0.413-0.460) |
| **New Hampshire** | 0.434(0.346-0.537) |
| **Hawaii** | 0.432(0.346-0.534) |
| **New Jersey** | 0.429(0.394-0.464) |
| **Alaska** | 0.423(0.295-0.589) |
| **North Dakota** | 0.392(0.282-0.532) |
| **Connecticut** | 0.390(0.340-0.440) |
| **District of Columbia** | 0.286(0.188-0.416) |
| **Total** | 0.538(0.531-0.544) |
